# 5-HT_2C_ Receptors in the Basolateral Amygdala and Dorsal Striatum Are a Novel Target for the Anxiolytic and Antidepressant Effects of Exercise

**DOI:** 10.1371/journal.pone.0046118

**Published:** 2012-09-25

**Authors:** Benjamin N. Greenwood, Paul V. Strong, Alice B. Loughridge, Heidi E. W. Day, Peter J. Clark, Agnieszka Mika, Justin E. Hellwinkel, Katie G. Spence, Monika Fleshner

**Affiliations:** 1 Department of Integrative Physiology, University of Colorado Boulder, Boulder, Colorado, United States of America; 2 Department of Psychology and Neuroscience, University of Colorado Boulder, Boulder, Colorado, United States of America; 3 The Center for Neuroscience, University of Colorado Boulder, Boulder, Colorado, United States of America; Hokkaido University, Japan

## Abstract

Physical activity reduces the incidence and severity of psychiatric disorders such as anxiety and depression. Similarly, voluntary wheel running produces anxiolytic- and antidepressant-like effects in rodent models. The specific neurobiological mechanisms underlying the beneficial properties of exercise, however, remain unclear. One relevant pharmacological target in the treatment of psychiatric disorders is the 5-HT_2C_ receptor (5-HT_2C_R). Consistent with data demonstrating the anxiogenic consequences of 5-HT_2C_R activation in humans and rodents, we have previously reported that site-specific administration of the selective 5-HT_2C_R agonist CP-809101 in the lateral/basolateral amygdala (BLA) increases shock-elicited fear while administration of CP-809101 in the dorsal striatum (DS) interferes with shuttle box escape learning. These findings suggest that activation of 5-HT_2C_R in discrete brain regions contributes to specific anxiety- and depression-like behaviors and may indicate potential brain sites involved in the anxiolytic and antidepressant effects of exercise. The current studies tested the hypothesis that voluntary wheel running reduces the behavioral consequences of 5-HT_2C_R activation in the BLA and DS, specifically enhanced shock-elicited fear and interference with shuttle box escape learning. After 6 weeks of voluntary wheel running or sedentary conditions, the selective 5-HT_2C_R agonist CP-809101 was microinjected into either the BLA or the DS of adult Fischer 344 rats, and shock-elicited fear and shuttle box escape learning was assessed. Additionally, in-situ hybridization was used to determine if 6 weeks of voluntary exercise changed levels of 5-HT_2C_R mRNA. We found that voluntary wheel running reduced the behavioral effects of CP-809101 and reduced levels of 5-HT_2C_R mRNA in both the BLA and the DS. The current data indicate that expression of 5-HT_2C_R mRNA in discrete brain sites is sensitive to physical activity status of the organism, and implicates the 5-HT_2C_R as a target for the beneficial effects of physical activity on mental health.

## Introduction

Physical activity is associated with a reduction in the incidence and severity of psychiatric disorders such as anxiety and depression [1], [2], [3], [4]. Similarly, voluntary exercise can reduce anxiety- [5], [6], [7], [8], [9], [10], [11] and depression- [8], [12], [13] like behaviors in laboratory rodents. Despite identification of many neuroadaptive changes produced by exercise [14], [15], [16], the specific neurobiological mechanisms underlying the anxiolytic and antidepressant properties of physical activity are unclear. Identification of these mechanisms could support the use of exercise as a prophylactic and therapeutic intervention for anxiety and depression, as well as provide insight into novel treatment strategies for psychiatric illness.

Serotonin (5-HT) has long been implicated in anxiety [17], [18], [19], [20], [21] and depression [22], [23], but the mechanisms by which 5-HT contributes to specific symptomatology of these disorders remain an intense topic of investigation. Emerging evidence points to a role for the 5-HT_2C_ receptor (5-HT_2C_R) in the expression of specific symptoms of anxiety [24], [25], [26] and depression [27], [28], [29]. Extending prior reports of anxiogenic effects of 5-HT_2C_R agonists in humans [30], [31], [32], [33], [34], [35], [36], [37] and rodents [38], [39], [40], [41], [42], [43], [44], our group has contributed to the identification of the specific brain regions that subserve specific behavioral effects of 5-HT_2C_R activation. Anxiety-like behavior, including exaggerated shock-elicited fear [45] and social avoidance [38], [41], can be elicited in rats by site-specific activation of 5-HT_2C_R in the region of the lateral/basolateral amygdala (BLA), a brain area classically implicated in fear and anxiety [46], [47]. In contrast, activation of 5-HT_2C_R in the dorsal striatum (DS) has no effect on anxiety behaviors, but can interfere with learning to escape in a shuttle box escape task, a common screening tool for antidepressant compounds [48]. Thus, 5-HT_2C_R activation in discrete brain regions appears to be a sufficient stimulus to elicit specific symptoms of anxiety- and depression-like behavior. Indeed, the social avoidance and shuttle box escape deficit produced by exposure to uncontrollable stress can be blocked by micro-injection of a selective 5-HT_2C_R antagonist into the lateral/BLA [49] and DS [50], respectively.

Despite the data implicating the 5-HT_2C_R as a potential target of anxiolytic and antidepressant strategies, knowledge regarding modulation of the 5-HT_2C_R by antidepressant drugs or environmental manipulation is limited. Chronic administration of the selective 5-HT reuptake inhibitor (SSRI) fluoxetine has been reported to increase site-specific 5-HT_2C_R pre-mRNA editing in the forebrain of BALB/c mice, an effect that would increase the pool of mRNA encoding 5-HT_2C_R with reduced function [51]. Chronic fluoxetine, however, had little effect on edited mRNA variants in C57BL/6 mice [51] or Sprague-Dawley rats [52]; whereas both chronic fluoxetine and reboxetine reduced the expression levels of non-edited 5-HT_2C_R mRNA in the prefrontal cortex of Sprague-Dawley rats [52]. These recent data suggest that one relevant effect of antidepressants could be reduced transcription of the 5-HT_2C_R. Similarly, prior evidence suggests that exercise may decrease sensitivity of 5-HT_2_ receptors in humans [53], [54] and rats [55]. Fox and colleagues, for example, found that voluntary exercise reduced the anxiogenic effect of the non-selective 5-HT_2_ receptor agonist metachlorophenylpiperazine on acoustic startle in mice [6]. Additionally, we have observed that the anxiety- and depression-like behavioral effects of acute administration of fluoxetine or uncontrollable stress, which depend upon activation of 5-HT_2C_R, can be prevented by 6 weeks of voluntary exercise [8], [9]. Therefore, one mechanism underlying the anxiolytic and antidepressant effects of exercise may be a reduction in the sensitivity and/or expression of the 5-HT_2C_R.

The current studies investigated whether the 5-HT_2C_R is a potential target for the anxiolytic and antidepressant effects of exercise. The 5-HT_2C_R agonist CP-809101 was microinjected either into the lateral/BLA to produce exaggerated fear, or into the DS to interfere with shuttle box escape behavior. We hypothesized that CP-809101 would produce exaggerated fear and interfere with shuttle box escape learning in a dose-dependent manner in sedentary rats, and that a higher dose of the 5-HT_2C_R agonist would be required to produce these behavioral effects in physically active rats. Additionally, *in situ* hybridization was used to investigate the effect of voluntary exercise on levels of 5-HT_2C_R mRNA in the amygdala and striatum.

## Methods

### Ethics Statement

All experimental protocols conformed to the NIH guide for the Care and Use of Laboratory Animals and were approved by the University of Colorado Institutional Animal Care and Use Committee protocol 1002.06. Care was taken to minimize animal discomfort during all procedures.

### Animals

Adult, male Fischer 344 rats (220–280 g at the time of behavioral testing; N = 165) were used in all experiments based on prior work optimizing 5-HT_2C_R-mediated behaviors in this strain [9], [45], [56]. The rats were housed in a temperature- (22°C) and humidity-controlled environment, were maintained on a 12∶12 hour light/dark cycle, and had *ad libitum* access to food and water. Rats assigned to the sedentary condition were individually housed in Nalgene Plexiglas cages (45×25.2×14.7 cm) lacking a running wheel. Rats assigned to 6 weeks of voluntary wheel running were individually housed in similar cages with attached running wheels. Animals were acclimated to these conditions for 1 week before any experimental manipulation. Wheels were rendered immobile with metal stakes during the acclimation period. Rats were weighed weekly.

### Voluntary wheel running

Rats (6–7 weeks of age) were randomly assigned to either remain sedentary or were allowed voluntary access to running wheels for 6 weeks, a duration of wheel running previously reported to prevent increased shock-elicited fear and deficits in shuttle box escape learning produced by exposure to uncontrollable stress [7], [8], [9]. At the start of each experiment, the wheels in the cages of the physically active rats were unlocked and these rats were allowed voluntary access to their wheels. Daily wheel revolutions were recorded digitally using Vital View software (Mini Mitter, Bend, OR, USA) and distance was calculated by multiplying wheel circumference (1.081 m) by the number of wheel revolutions.

### Surgery

Rats underwent surgery during the 4^th^ week of either voluntary wheel running or sedentary conditions. Under ketamine (0.75 mg/kg i.p.; Vedco, St. Joseph, MO, USA) and medetomidine (0.5 mg/kg i.p.; Pfizer, New York, NY, USA) anesthesia, bilateral cannulae (26 gauge, Plastics One, Roanoke, VA, USA) were implanted as previously described [45]. Cannulae were aimed at either the DS: +0.5 A/P, ±3.0 M/L, and −4.6 D/V from bregma [45], [57] or, separately, the region of the lateral/BLA: −3.0 A/P, ±4.8 M/L, −6.2 D/V from bregma [41], [45], based on the atlas by Paxinos and Watson [58]. Atipamezole (0.5 mg/kg i.p.; Pfizer, New York, NY, USA) was administered following surgery to reverse the effects of medetomidine. All rats were inoculated with 0.25 mL/kg (subcutaneous) penicillin (Combi-Pen, Agrilabs, St. Joseph, Missouri, USA) immediately following surgery and returned to their home cages. Behavioral experiments were conducted approximately 2–3 weeks after implantation surgery, by which time running behavior had returned to pre-surgical levels. Following experiment completion, brains were sliced at 40 µm and stained with Cresyl Violet for cannulae placement verification. Misplaced cannulae were excluded from analysis or used as off-site controls when appropriate.

### Drug Microinjections

Microinjections of the selective 5-HT_2C_R agonist CP-809191 (Tocris Bioscience, Ellisville, MO, USA) were made 15 minutes prior to behavioral testing. On the day of behavioral testing, a micro-injector extending 0.5 mm (intra-DS) or 1.0 mm (intra-BLA) beyond the tip of the guide cannula was inserted. Drug doses and injection volumes were based on prior work examining the behavioral effects of CP-809101 [41], [45], [56]. Specifically, CP-809191 was dissolved in 0.9% sterile saline and gently warmed at 45°C for 10–15 minutes in a water bath at concentrations of 0.3 mM, 2.0 mM and 6.0 mM. CP-809101 was administered intra-DS at a volume of 1.0 µL/side or intra-BLA at a volume of 0.5 µL/side [41], [45]. Rats receiving both CP-809101 along with a 5-HT_2C_R antagonist were not included in the current study because we have previously shown that the behavioral effects of 6 mM CP-809101 can be blocked by pretreatment with the selective 5-HT_2C_R antagonist SB242084 when the drugs are injected either systemically [56] or intra-DS [45].

### Behavioral Testing

Behavioral testing was conducted following 6 weeks of voluntary wheel running or sedentary conditions, 15 minutes following drug microinjections. Fear and shuttle box escape behaviors were assessed sequentially in shuttle boxes (50.8 cm×25.4 cm×30.48 cm, Coulbourn Instruments, Whitehall, PA) using procedures previously described [8], [45], [59]. At the beginning of each session, rats were placed into shuttle boxes and allowed to explore for 10 minutes. During this 10 minute period (pre-shock period), fear behavior was assessed using a sampling procedure in which each rat was scored every 10 seconds as either freezing, defined as an absence of all movement except for that required for respiration, or not freezing. Spontaneous shuttle box crosses were also counted during this time in a subset of rats (N = 3–8/group) as a measure of locomotor activity in response to a novel environment. Rats then received two 0.7 mA foot shocks (1 minute ITI) delivered through both sides of the grid floor. Foot shocks were terminated when the rat fully crossed over to the opposite side of the shuttle box (fixed ratio 1, FR-1). The latencies to cross were recorded (FR-1 latencies). Following the second FR-1 trial, shock-elicited freezing was observed for 20 minutes. Shock-elicited freezing is a measure of fear conditioned to cues present in the shuttle box [60]. Exaggerated shock-elicited fear has been argued to represent anxiety [61]. The post shock freezing period was followed by 25 fixed-ratio 2 (FR-2) escape trials (average ITI of 1 min). During FR-2 trials, rats were required to cross through the shuttle box door twice in order to terminate the foot shock (0.6 mA). An escape latency of 30 seconds was assigned if a correct escape response did not occur within 30 seconds, at which time the shock was terminated. A single test session lasted approximately 1 hour and occurred between 0900 and 1200. All animals were scored for both freezing and escape behavior by an experimenter blind to treatment condition of the animals.

### In Situ Hybridization

In a separate experiment, rats were randomly assigned to either remain sedentary or allowed voluntary access to running wheels for 6 weeks. After 6 weeks, rats were sacrificed via rapid decapitation. Following previously published *in situ* hybridization protocols [8], [62], [63], brains were extracted, and frozen in isopentane cooled with dry ice (−20°C; 4 minutes). Brains were stored at −80°C prior to being sectioned at 10 µm thickness with a cryostat. Slicing occurred at −21°C, and rostral-caudal sections of the DS and BLA were collected and thaw-mounted onto poly-L-lysine-coated slides. Tissue sections were stored at −80°C prior to use in single-label radioactive *in situ* hybridizations. Before hybridization, sections were fixed in 4% paraformaldehyde for 1 hour, washed 3 times in 2× sodium saline citrate (SSC), acetylated with 0.25% acetic anhydride containing 0.1 M triethanolamine for 10 minutes, and dehydrated in graded ethanol. The 5HT_2C_R receptor plasmid construct was obtained from Dr. David Julius at the University of California, San Francisco. The 5HT_2C_R probe is 555 base pairs long, spanning 1370–1925 (Accession # M21410). Customary transcription protocols were used to label the 5HT_2C_R riboprobe with ^35^S-UTP. Following completion of transcription, the riboprobe was mixed with 50% hybridization buffer comprised of 50% high-grade formamide, 10% dextran sulfate, 3× SSC, 1× Denhardt's solution, 0.2 mg/mL yeast tRNA, and 0.05 M sodium phosphate (pH 7.4). The 5HT_2C_R riboprobe in hybridization buffer was applied directly to slides containing sections of DS and BLA. Slides were incubated overnight at 55°C in humid chambers. The following day, slides were washed 3 times in 2× SSC, subjected to an RNase A (200 µg/ml) treatment for 1 hour, rinsed in graded concentrations of SSC, washed in 0.1× SSC (65°C) for 1 hour, and dehydrated in ethanol. After drying, slides were placed in light-tight autoradiography cassettes, and exposed to X-ray film (Biomax-MR) for 1 week.

### Image Analysis for In Situ Hybridization

Levels of 5-HT_2C_R mRNA were analyzed by computer-assisted optical densitometry following previously published protocols [62], [63]. Brain section images were captured digitally (CCD camera, model XC-77; Sony, Tokyo, Japan), and the relative optical density of the x-ray film was determined using Scion Image Version 4.0 (Scion, Frederick, MD, USA). A macro was written that enabled signal above background to be determined automatically. For each section, a background sample was taken over an area of white matter, and the signal threshold was calculated as mean gray value of background +3.5 standard deviations. The section was automatically density-sliced at this value, so that only pixels with gray values above these criteria were included in the analysis. Results are expressed as mean integrated density, which reflects both the signal intensity and the number of pixels above assigned background (mean signal above background×number of pixels above background). Each subject's mean integrated density at a given level represents the average of at least 2 slices chosen for analysis between the following coordinates: Striatum from +1.60 to +0.20 mm anterior to bregma; amygdala and lateral ventricle from −2.56 to −3.30 mm posterior to bregma based on the atlas by Paxinos and Watson [58]. Templates for each region were made to ensure that equivalent areas were analyzed between animals. Lateral ventricle was analyzed as a control region because the choroid plexus of the lateral ventricle expresses relatively large amounts of 5-HT_2C_R mRNA.

### Statistical Analysis

Body weights were analyzed with repeated measures ANOVA. Pre-shock freezing scores were averaged into 1 pre-shock score and analyzed with ANOVA. Shock-elicited freezing scores were collapsed into 10, 2 min blocks and analyzed using 2×3 (activity×drug), repeated measures ANOVA. The two FR-1 trials were averaged into a single FR-1 latency score and analyzed with ANOVA. FR-2 escape latencies were averaged into 5 blocks of 5 trials each and analyzed with 2×3 (activity×drug), repeated measures ANOVA. Average post-shock freezing and average FR-2 escape latencies were analyzed with ANOVAs that included an additional group of off-site controls when appropriate. Group differences in 5-HT_2C_R mRNA expression in the medial and lateral DS, lateral amygdala, BLA, central amygdala (CeA), and lateral ventricle were analyzed with ANOVA. Tukey-Kramer post hoc analyses were performed when appropriate. Results were considered significant when p≤0.05.

## Results

### Voluntary exercise reduces exaggerated fear produced by 5-HT_2C_R activation in the BLA

**Figure 1:**
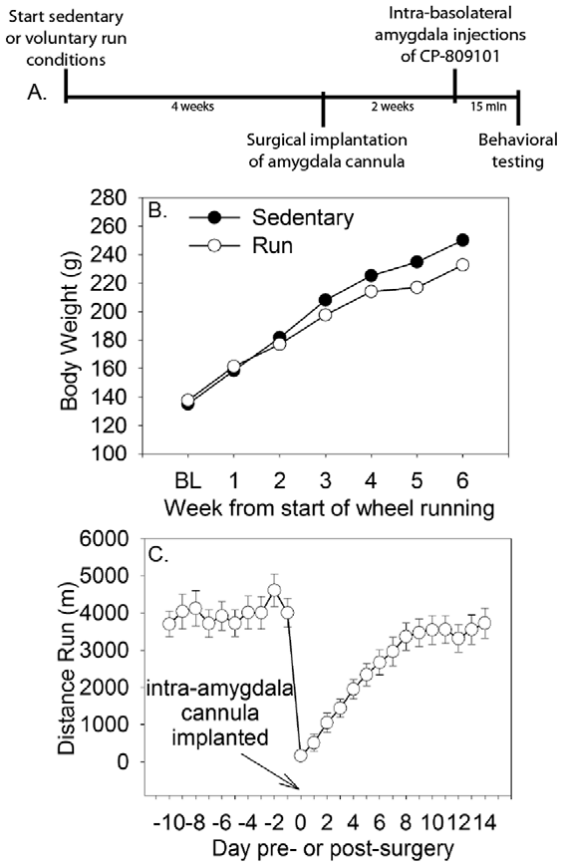
Intra-amygdala experimental design, body weights and running distance. (**A**) Experimental timeline. Adult, male Fischer 344 rats were allowed voluntary access to running wheels for 6 weeks (Run) or remained sedentary. All rats had cannulae implanted into the region of the lateral/basolateral amygdala (BLA) between weeks 3 and 4. Three weeks later, rats were injected with either saline or increasing doses of the selective 5-HT_2C_ receptor agonist CP-809101 (0.3 mM, 2.0 mM, or 6.0 mM) through the guide cannulae. Rats were placed into shuttle boxes 15 minutes following intra-DS injections and shock-elicited freezing and escape learning were tested sequentially. (**B**) Mean weekly body weight (grams) of physically active and sedentary rats. (**C**) The daily distance run (meters) pre- and post- cannula implantation surgery. Data represent means ± SEM.

An experimental timeline is shown in Figure 1A. Sedentary and physically active rats weighed similar amounts at the beginning of the study, but physically active rats gained less weight over the course of the experiment (Figure 1B; (F (6, 480) = 16.03; p<0.0001). Intra-BLA cannulae were surgically implanted after 4 weeks of voluntary wheel running. Figure 1C shows the average daily distance run pre- and post-surgery. As expected, surgery immediately decreased running distance. However, running distance resumed quickly and continued to steadily increase during the week after surgery.

**Figure 2:**
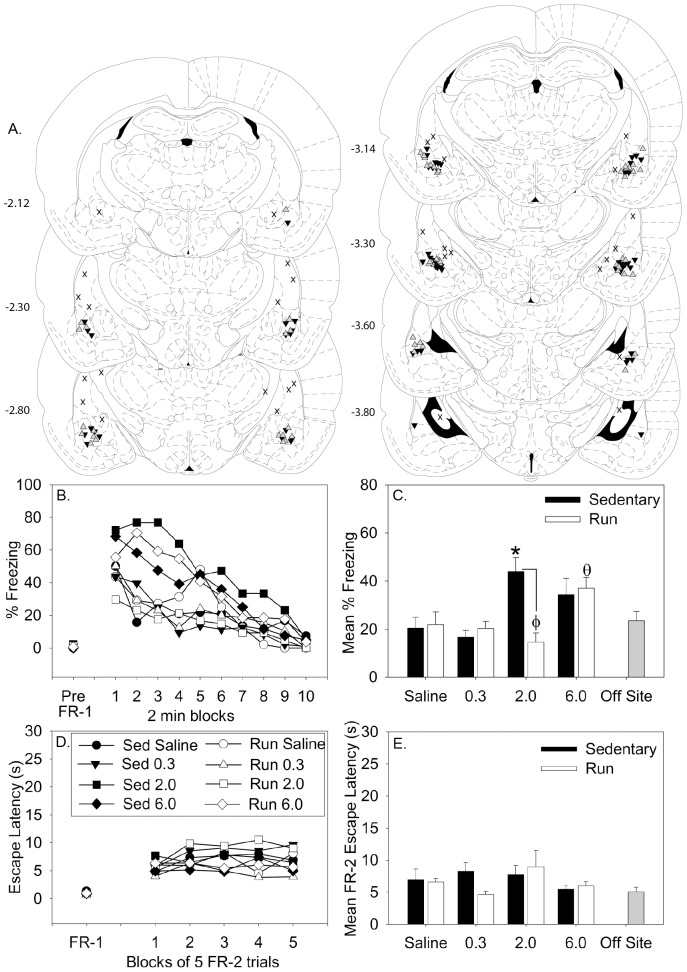
Six weeks of voluntary wheel running reduces the increase in fear behavior produced by 5-HT_2C_ receptor activation in the region of the lateral/basolateral amygdala (BLA). Following 6 weeks of voluntary wheel running (Run) or no running (Sedentary), rats received intra-BLA microinjections of either saline or the selective 5-HT_2C_ receptor agonist CP-809101 (0.3 mM, 2.0 mM, or 6.0 mM). Shockelicited freezing and shuttle box escape latency were measured sequentially in shuttle boxes 15 minutes later. (**A**) Cannula placement within the amygdala. Sedenatry rats are denoted with black triangles, physically active rats are denoted with gray triangles, and off-site placements are denoted with an **X**. Brain illustrations adapted from Paxinos and Watson (published in the Rat Brain in Stereotaxic Coordinates, 4^th^ ed., Copyright Elsevier (1998)). Numbers left of illustrations refer to distance from Bregma (mm). (**B**) Mean freezing behavior presented in 2 minute blocks (pre-FR-1 scores are not different and therefore overlap). Error bars are ommited for clarity. (**C**) The mean percent shock-elicited freezing for the entire 20 minute observation period. (**D**) Shuttle box escape latencies for one block of 2 FR-1 trials (FR-1) and five blocks of 5 FR-2 trials (FR-2). Error bars are omitted for clarity. (**E**) The mean escape latency for all 25 FR-2 escape trials. Data represent group means ± SEM. * p<0.05 relative to Offsite Control, Sedentary/Saline, Sedentary/0.3 mM, Run/2.0 mM, and Run/6.0 mM groups. Φ p<0.05 relative to the Sedentary/2.0 mM group. θ p<0.05 relative to the Run/2.0 mM group.

A graphical representation of cannula placements is shown in Figure 2A. After exclusion of rats with misplaced cannula (i.e. cannulae tips were outside of the lateral amygdala/BLA), group sizes were as follows: Sedentary/Saline = 8; Sedentary/0.3 mM = 8; Sedentary/2.0 mM = 10; Sedentary/6.0 mM = 10; Run/Saline = 4; Run/0.3 mM = 8; Run/2.0 mM = 9; Run/6.0 mM = 9. Data obtained from rats with misplaced cannulae (Sedentary/2.0 mM = 2; Sedentary/6.0 mM = 4; Run/2.0 mM = 5; Run/6.0 mM = 5) were averaged and included as an off-site control group.

Consistent with prior reports that wheel running reduces general locomotor activity [64], [65], physically active rats (9.05±1.4 crossings) performed significantly fewer (F (1, 31) = 15.62; p = 0.0004) shuttle box crossings during the 10 min pre-shock observation period compared to their sedentary counterparts (12.63±0.98 crossings), regardless of drug condition. It is therefore unlikely that an increase in general locomotor activity contributed to any observed behavioral effects of exercise. Voluntary exercise increased the intra-BLA dose of CP-809101 necessary to produce an increase in shock-elicited freezing behavior. Whereas 2.0 mM CP-809101 injected into the BLA increased shock-elicited fear in sedentary rats, 6.0 mM CP-809101 was required to enhance fear in physically active rats. The effect of the 5-HT_2C_R agonist CP-809101 on freezing behavior over the course of the 20 minute freezing observation period is shown in Figure 2B. Pre-shock freezing was minimal and did not differ between groups (Figure 2B, Pre-FR-1). The main effects of drug (F (3, 57) = 5.612; p = 0.002) and time (F (9, 513) = 39.6; p<0.0001), as well as the interaction between exercise and drug (F (3, 57) = 7.187; p = 0.0004), were all significant. Post hoc comparisons revealed that the Sedentary/2.0 mM group differed from the Sedentary/Saline group during the 2^nd^ and 3^rd^ freezing blocks, from the Sedentary/0.3 mM and Run/0.3 mM groups during the 3^rd^ and 4^th^ freezing blocks, and from the Run/2.0 mM group during the 1^st^, 2^nd^, and 3^rd^ freezing blocks. The Sedentary/6.0 mM group differed from the Sedentary/Saline group during the 2^nd^ freezing block. The Run/6.0 mM group differed from the Run/2.0 mM group during the 2^nd^ and 3^rd^ freezing block. At no time did the 0.3 mM groups differ from the Saline groups. Average time spent freezing is shown in Figure 2C and escape behavior is shown in Figures 2D and 2E. Consistent with our prior observations, neither exercise [7], [8] nor activation of the 5-HT_2C_R in the BLA [50] prior to behavioral testing altered FR-1 or FR-2 escape behavior.

### Voluntary exercise reduces shuttle box escape deficit produced by 5-HT_2C_R activation in the DS

**Figure 3:**
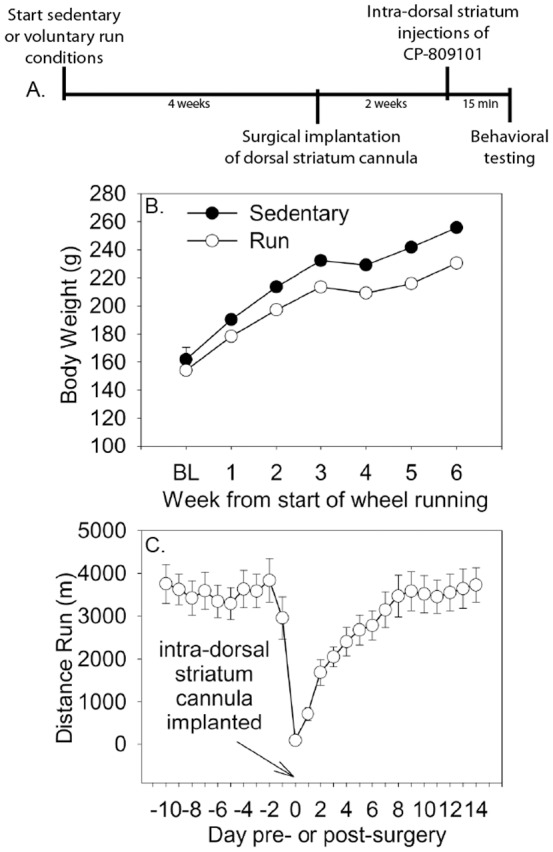
Intra-striatum experimental design, body weights and running distance. (**A**) Experimental timeline. Adult, male Fischer 344 rats were allowed voluntary access to running wheels for 6 weeks (Run) or remained sedentary. All rats had cannulae implanted into the region of the dorsal striatum (DS) between weeks 3 and 4. Three weeks later, rats were injected with the selective 5-HT_2C_ receptor agonist CP-809101 (0.3 mM, 2.0 mM, or 6.0 mM) through the guide cannulae. Rats were placed into shuttle boxes 15 minutes following intra-DS injections and shock-elicited freezing and escape learning were tested sequentially. (**B**) Mean weekly body weight (grams) of physically active and sedentary rats. (**C**) The daily distance run (meters) pre- and post- cannula implantation surgery. Data represent group means ± SEM.

An experimental timeline is shown in Figure 3A. Sedentary and physically active rats weighed similar amounts at the beginning of the study, but physically active rats gained less weight over the course of the experiment (Figure 3B; F (6, 402) = 7.229; p<0.0001). Intra-DS cannulae were surgically implanted during the 4^th^ week of voluntary wheel running. Figure 3C shows the average daily distance run pre- and post-surgery. Running behavior resumed quickly after surgery and continued to steadily increase during the following week to levels observed prior to surgery.

**Figure 4:**
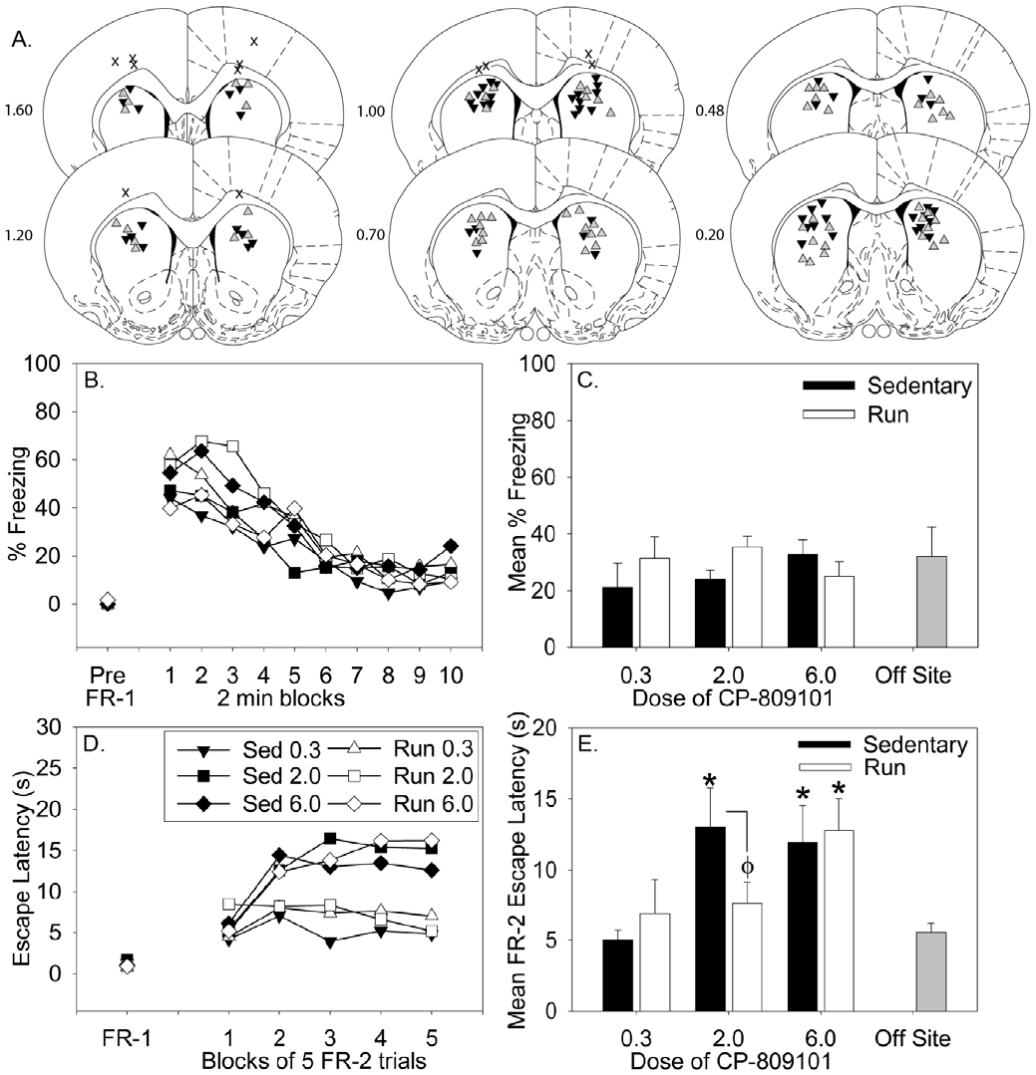
Six weeks of voluntary wheel running reduces the deficit in instrumental escape learning produced by 5-HT_2C_ receptor activation in the dorsal striatum. Fifteen minutes prior to behavioral testing, sedentary and physically active (Run) rats received intra-DS microinjections of the 5-HT_2C_ receptor agonist CP-809101 (0.3 mM, 2.0 mM or 6.0 mM). (**A**) Cannula tip placement within the DS. Sedentary rats are denoted with black triangles, physically active rats are denoted with gray triangles, and off-site placements are denoted with an **X**. Brain illustrations adapted from Paxinos and Watson (published in the Rat Brain in Stereotaxic Coordinates, 4^th^ ed., Copyright Elsevier (1998)). Numbers left of illustrations refer to distance from Bregma (mm). (**B**) Freezing behavior over the duration of the post-FR-1 freezing observation period presented in 2 minute blocks (pre-shock scores are not different and therefore overlap). (**C**) The mean percent time spent freezing during the 20 minute observation period. (**D**) Shuttle box escape latencies for one block of 2 FR-1 trials (FR-1) and five blocks of 5 FR-2 trials (FR-2). (**E**) The mean escape latency for all 25 FR-2 escape trials. Data represent group means ± SEM. * p<0.05 relative to 0.3 mM groups and off-site control group; Φ p<0.05 relative to 2.0 mM sedentary group.

Intra-DS cannula placements are shown in Figure 4A. After exclusion of rats with misplaced cannulae, group sizes were Sedentary/0.3 mM = 7; Sedentary/2.0 mM = 12; Sedentary/6.0 mM = 11; Run/0.3 mM = 9; Run/2.0 mM = 15; Run/6.0 mM = 9. Data from rats with misplaced cannulae (Sedentary/2.0 mM = 2; Sedentary/6.0 mM = 1; Run/6.0 mM = 3) were averaged and included as off-site controls. Saline was not injected into the DS in this study because our results from the above amygdala study, as well as our prior studies [50], indicate that saline injected animals behave identically to animals injected with 0.3 mM CP-809101.

The effect of the 5-HT_2C_R agonist CP809101 on freezing blocks and average freezing behavior are shown in Figure 4B and Figure 3C, respectively. Pre-shock freezing was minimal and did not differ between groups (Figure 4B, pre-FR-1). While there was an expected effect of time (F (9, 513) = 36.546; p<0.0001) on freezing behavior during freezing blocks, neither physical activity status nor intra-DS administration of CP-809101 prior to behavioral testing impacted freezing behavior.

ANOVA revealed a significant main effect of exercise on FR-1 behavior (p = 0.003), i.e. exercise groups had faster average FR-1 escape latencies than sedentary groups. However, neither the main effect of drug nor the interaction between exercise and drug were significant (Figure 4D, FR-1). Repeated measures ANOVA revealed significant main effects of time (F (4, 228) = 16.919; p = <0.0001) and drug (F (2, 57) = 3.476; p = 0.0376), as well as reliable time×drug (F (4, 228) = 2.384; p = 0.0174) and time×drug×exercise (F (8, 228) = 3.232; p = 0.0017) interactions on FR-2 escape blocks (Figure 4D). Average FR-2 escape times are shown in Figure 4E. Post hoc analysis revealed that there was no difference between exercise and sedentary animals that received the sub-threshold dose of CP-809101 (0.3 mM). Importantly, the 2.0 mM dose of CP-809101 administered into the DS interfered with shuttle box escape learning in sedentary rats only, whereas physically active rats were resistant to the behavioral effects of that dose. Finally, the highest dose of CP-809101 (6.0 mM) was sufficient to interfere with escape learning in both sedentary and physically active rats. Off-site control rats behaved similarly to the 0.3 mM groups.

### Voluntary exercise decreases levels of 5-HT_2C_R mRNA in the amygdala and dorsal striatum

**Figure 5:**
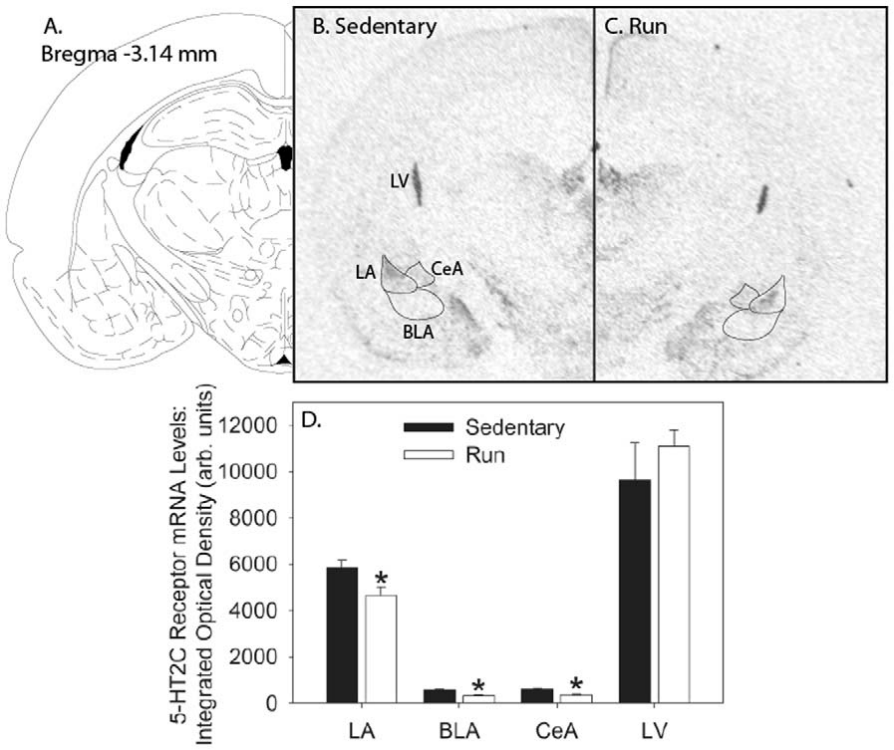
Six weeks of voluntary wheel running decreases 5-HT_2C_ mRNA expression in the amygdala. (**A**) The region of the amygdala as shown by Paxinos and Watson (published in the Rat Brain in Stereotaxic Coordinates, 4^th^ ed., Copyright Elsevier (1998)). (**B**) Representative autoradiographic coronal section through the region of the striatum (Bregma – 3.14 mm) in a sedentary rat processed with *in situ* hybridization for 5-HT_2C_R messenger ribonucleic acid (mRNA). (**C**) Representative autoradiographic coronal section through the region of the amygdala (Bregma – 3.14 mm) in a physically active rat (Run) processed with *in situ* hybridization for 5-HT_2C_R mRNA. (**D**) Relative levels of 5-HT_2C_ receptor (mRNA) in the lateral amygdala (LA), basolateral amygdala (BLA), central amygdala (CeA), and lateral ventricle (LV) of sedentary rats or rats allowed voluntary access to running wheels for 6 weeks (Run). Values represent mean integrated density ± SEM. * p≤0.05 relative to respective sedentary groups.

**Figure 6:**
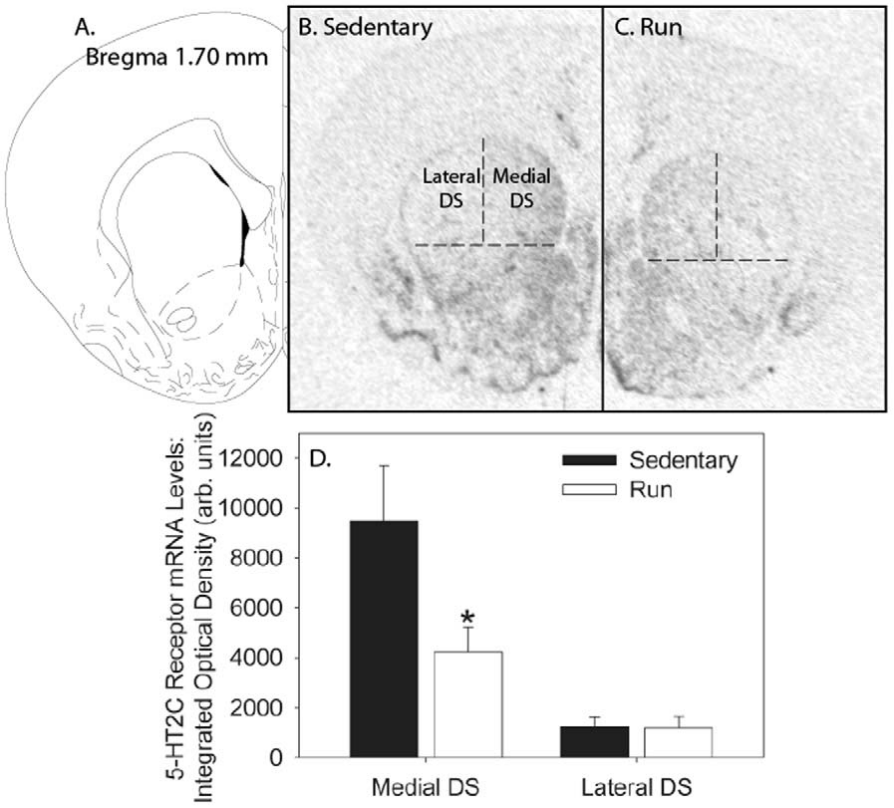
Six weeks of voluntary wheel running decreases 5-HT_2C_ mRNA expression in the medial dorsal striatum. (**A**) The region of the striatum as shown by Paxinos and Watson (published in the Rat Brain in Stereotaxic Coordinates, 4^th^ ed., Copyright Elsevier (1998). (**B**) Representative autoradiographic coronal section through the region of the striatum (Bregma 1.70 mm) in a sedentary rat processed with *in situ* hybridization for 5-HT_2C_R messenger ribonucleic acid (mRNA). (**C**) Representative autoradiographic coronal section through the region of the striatum (Bregma 1.70 mm) in a physically active rat (Run) processed with *in situ* hybridization for 5-HT_2C_R mRNA. (**D**) Relative levels of 5-HT_2C_ receptor (mRNA) in the medial and lateral dorsal striatum (DS) of sedentary rats or rats allowed voluntary access to running wheels for 6 weeks (Run). Values represent mean integrated density ± SEM. * p≤0.05 relative to respective sedentary group.

Non-surgerized rats naïve to behavioral testing were allowed voluntary access to running wheels (N = 7) or remained sedentary (N = 7) for 6 weeks to determine the effects of voluntary exercise on 5-HT_2C_ mRNA levels. Weight and running data were similar to prior studies (data not shown). Representative autoradiographs showing 5-HT_2C_R mRNA in the amygdala and striatum of sedentary and physically active rats are shown in Figure 5 and Figure 6, respectively. Relative to sedentary rats, 6 weeks of wheel running reduced levels of 5-HT_2C_R mRNA in the lateral amygdala (F (1, 12) = 6.95; p = 0.02), BLA (F (1, 12) = 25.71; p = 0.0003), and central amygdala (F (1, 12) = 12.89; p = 0.004), but not the lateral ventricle (F (1, 12) = 0.705; p = 0.42), where expression of 5-HT_2C_R mRNA was most pronounced (Figure 5D). Wheel running also reduced 5-HT_2C_R mRNA levels in the medial DS (F (1, 12) = 4.66; p = 0.05), but not the lateral DS (F (1, 12) = 0.003; p = 0.95; Figure 6D).

## Discussion

The current data demonstrate for the first time that exercise can reduce anxiety-like behaviors produced by administration of a selective 5-HT_2C_R agonist into discrete brain regions, and implicate 5-HT_2C_R in the BLA and DS as a potential target for the anxiolytic and antidepressant properties of exercise. Specifically, 6 weeks of voluntary wheel running increased the dose of intra-BLA and -DS CP-809110 necessary to produce exaggerated fear and interference with escape learning, respectively. *In-situ* hybridization revealed that voluntary wheel running decreased the levels of 5-HT_2C_R mRNA in brain regions implicated in these behaviors, including the BLA and the DS. These data add to our understanding of the neural pathways and mechanisms underlying the psychological and behavioral benefits associated with regular physical activity.

Prior work has shown that 5-HT_2C_R agonist injections into the BLA increase anxiety-like behavior in rodents [38], [41], [42], [45]. Here we report that 6 weeks of voluntary wheel running was sufficient to reduce the exaggerated fear produced by activation of 5-HT_2C_R in the lateral/BLA. Physical activity, therefore, may reduce the expression of some anxiety-like behaviors through a reduction in the expression, sensitivity or function of 5-HT_2C_R in the lateral/BLA. Additionally, voluntary wheel running reduced levels of 5-HT_2C_R mRNA in the lateral amygdala and BLA, suggesting that physical activity may reduce the behavioral consequences of 5-HT_2C_R agonist administration via a reduction in transcription of 5-HT_2C_R. Interestingly, voluntary wheel running also reduced 5-HT_2C_R mRNA in the CeA, another area implicated in fear behavior [66], [67]. The role of the 5-HT_2C_R in the CeA in anxiety, however, remains relatively unknown. 5-HT_2C_Rs are expressed throughout the amygdala complex, but 5-HT_2C_R mRNA (Figure 5), as well as receptor density [68], appears to be greatest in the lateral amygdala. Moreover, activation of 5-HT_2C_R in the CeA has no effect on the expression of some types of anxiety-like behaviors [38], [41]. Instead, it appears that anxiety-like effects of 5-HT activity in the CeA are more likely mediated by 5-HT_1A_ receptors [69]. Further work is necessary to determine if the observed reduction of 5-HT_2C_R mRNA levels in the CeA of physically active rats contributes to a behavioral effect of exercise.

In addition to the BLA, the current data implicate the 5-HT_2C_R in the DS as a target for the antidepressant effects of exercise. Hypoactivity of striatal dopaminergic neurotransmission is hypothesized to occur in depression [70], [71]. Consistent with this idea, the shuttle box escape deficit produced by uncontrollable stress (an established animal model of depression) can be thought of as a failure of a rapid instrumental learning process [50] which requires dopamine in the DS for optimal acquisition [72]. Importantly, extracellular dopamine in the DS can be reduced by DS 5-HT_2C_R activation [73]. It is therefore possible that the reduced expression and function of 5-HT_2C_R in the DS of physically active, relative to sedentary, rats observed in the current study could alleviate depressive symptoms by restoring dopamine transmission in the DS. Indeed, exercise also prevents the shuttle box escape deficit produced by uncontrollable stress [8], [74].

Although cannulae in the current study were aimed at the border between the medial and lateral DS, it is likely that 5-HT_2C_R activation interfered with escape behavior through action in the medial portion of the DS. 5-HT_2C_R are expressed more heavily in the medial, relative to the lateral, DS ([75] and Figure 6). Moreover, it is widely accepted that distinct sub-regions of the DS mediate different aspects of instrumental learning. The early stages of instrumental learning are modulated by the medial DS, whereas the lateral DS contributes to the later stages of instrumental learning (for review see [76], [77]). The fact that the acquisition of the escape contingency by rats injected with the low dose of CP-809101 occurs rapidly (within the first 5 trials), seems to indicate a role for the medial DS (see also [50]). Finally, voluntary exercise reduced 5-HT_2C_R mRNA in specifically the medial, and not the lateral, DS. These data support a role for 5-HT_2C_R activation in the medial DS in interference with shuttle box escape behavior and aversively-motivated instrumental learning in general.

It is important to note that the 5-HT_2C_R is known to undergo post-translational modifications. Increasing evidence suggests that the editing of 5-HT_2C_R mRNA can lead to the expression of multiple 5-HT_2C_R isoforms that have different G-protein activity and affinities for 5-HT [78], [79], altered constitutive activity [80], [81], as well as intracellular effects [82]. These editing-induced changes in 5-HT_2C_R function have been speculated to play a critical role in the etiology of anxiety and depression [83], [84], [85], [86]. Additionally, 5-HT_2C_R mRNA editing changes seem to occur after perturbations of 5-HT levels. Specifically, persistent increases in 5-HT neurotransmission, via drugs or SERT gene deletion, increase the occurrence of 5-HT_2C_R mRNA editing events, while at the same time decreasing 5-HT_2C_R responsiveness [52], [87], [88]. Interestingly, acute bouts of physical activity (albeit forced) can also increase central 5-HT [89]. It is possible that there is an overlap between some of the underlying mechanisms of therapeutic drugs and exercise, such that persistent changes in 5-HT neurotransmission over extended periods of time, whether due to a regular drug regimen or exercise program, can produce long-term plastic changes in the function of post-synaptic modulators of behavior such as the 5-HT_2C_R. In addition to changing mRNA levels in discrete brain regions, exercise may also affect 5-HT_2C_R pre-mRNA editing events, which could contribute to our observed reduction in the expression of anxiety- and depression-like behaviors. The effect of regular voluntary exercise on the editing of 5-HT_2C_R mRNA needs to be further explored.

In conclusion, physical activity reduces anxiety- and depression-like behaviors produced by 5-HT_2C_R activation in discrete brain regions. The current data extend previous work identifying the 5-HT_2C_R as a relevant target for pharmacological discovery, as well as shed light on potential mechanisms which underlie the anxiolytic effects of physical activity. Finally, our results implicate physical activity as one environmental variable with the ability to influence transcription or sensitivity of the 5-HT_2C_R. Future studies could therefore utilize exercise as a tool to reveal novel means with which to modulate expression of the 5-HT_2C_R in discrete brain regions relevant to stress-related psychiatric disorders.
